# Origin of scarlet gynogenetic triploid *Carassius* fish: Implications for conservation of the sexual–gynogenetic complex

**DOI:** 10.1371/journal.pone.0276390

**Published:** 2022-10-20

**Authors:** Tappei Mishina, Kazuhiro Nomoto, Yoshiyasu Machida, Tsutomu Hariu, Katsutoshi Watanabe

**Affiliations:** 1 Laboratory of Animal Ecology, Graduate School of Science, Kyoto University, Sakyo-ku, Kyoto, Japan; 2 Laboratory for Chromosome Segregation, RIKEN Center for Biosystems Dynamics Research, Chuo-ku, Kobe, Japan; 3 Kushiro City Museum, Kushiro, Hokkaido, Japan; 4 Bihoro Museum, Bihoro, Hokkaido, Japan; 5 Kushiro Public University of Economics, Kushiro, Hokkaido, Japan; National Cheng Kung University, TAIWAN

## Abstract

Conservation of sperm-dependent asexual (gynogenetic) species is challenging due to their complicated ecological dynamics, which requires the stable coexistence with their sperm-providing sexual relatives, who often share similar niches. A symbolic but vulnerable gynogenetic animal is the scarlet *Carassius* fish, or Hibuna, which is mainly found in Lake Harutori on Hokkaido, Japan. Although Hibuna in Lake Harutori has been protected as a symbol of the Natural Monument of Japan, it has recently suffered population decline. To establish an effective conservation strategy for Hibuna, we investigated its origin, reproductive mode, and genetic diversity, with reference to the surrounding wild populations, using nuclear microsatellites and mitochondrial gene sequences. Our genetic analyses revealed that the main ploidy of Hibuna was triploid or tetraploid, and it reproduces gynogenetically. However, no co-existing sexual diploid *Carassius* was detected among our samples, suggesting that the sexual diploids and the gynogenetic population including Hibuna would be at risk of co-extirpation. In addition, Hibuna showed high genetic/clonal diversity and most Hibuna had nonindigenous mitochondrial haplotypes that are mostly identical to those reported from goldfish. These results indicate that Hibuna most probably originated from hybridization between indigenous gynogenetic triploids and goldfish introduced about 100 years ago, involving rare sexual reproduction. This spontaneous long-term field experiment exemplifies the recently documented diversification process of gynogenetic *Carassius* via complex interploidy gene flow. Although the priority to be placed on the conservation of Hibuna is controversial, the maintenance of gynogenetic *Carassius*, including Hibuna, requires strategic conservation of sexual populations.

## Introduction

The reproductive mode (e.g., outcrossing, selfing, and asexual reproduction) of protected species is one of the key determinants of genetic diversity that affects the effective population size (Ne) and selection dynamics [1, 2]. Gynogenesis is an example of an extreme form of asexual reproduction, but has attracted attention in the field of evolution [3, 4]. During gynogenesis, egg development must be stimulated by sperm donated from a close bisexual species; however, the sperm nucleus degenerates without fusing with the egg nucleus, resulting in no genetic contribution to the offspring being made [5]. Gynogenesis is considered to be associated with a risk of extinction and increases the complexity of managing endangered species due to their highly complicated ecological dynamics [4]. A short-term effect of gynogenesis is that it can result in a rapid increase in the population because gynogenesis does not need to produce males, known as the two-fold cost of sex [6]. However, this short-term advantage of gynogenesis can either extirpate the sexual relatives, which are essential for the reproduction of gynogenetic species, or lead to coextinction as both gynogenetic and sexual populations share a similar niche [4]. In contrast, the long-term effect of gynogenesis has been shown to be associated with the risk of extinction due to the loss of genetic recombination, which has several disadvantages including restricted adaptability to changing environments, limited resistance to disease, and the accumulation of deleterious mutations [6–8]. However, recent studies have shown that the limitations of gynogenetic species can be attenuated through the acquisition of genetic diversity via introgressed genome segments from their sexual sperm donor relatives through various mechanisms, such as kleptogenesis [9, 10] and rare sexual reproduction [11]. Thus, for the appropriate management of gynogenetic species, three key aspects should be considered: (i) protecting both gynogenetic species and their sexual relatives, (ii) estimating and maintaining genetic/clonal diversity, and (iii) understanding the mechanisms by which genetic diversity is increased among gynogenetic populations.

The gynogenetic triploid *Carassius* fish is interesting subject for studying evolution and conservation of asexual vertebrates. There has been taxonomical confusion in this genus, but currently at least three species are recognized: the crucian carp (*Carassius carassius*), the Japanese white crucian carp (*C*. *cuvieri*), and the wild goldfish, *C*. *auratus*-complex (sometimes referred such as *C*. *gibelio*, *C*. *langsdorfii*, and *C*. *buergeri* [12–14]), which is distributed in East to Northeast Asian watersheds. Several *C*. *auratus*-complex populations have been reported to have decreased in size over the years and are currently regarded as endangered [15, 16]. However, efforts to conserve this species complex have been hindered by taxonomic confusion and ploidy variation (sexual diploid, gynogenetic triploid, and rare tetraploid) [17, 18]. The gynogenetic triploids of *C*. *auratus*-complex produce offspring parthenogenetically, yet egg development cannot be completed without insemination by sperm from a sexual diploid *Carassius*, which are discarded during development [17].

Among the *C*. *auratus*-complex, the populations attracting particular attention are those including scarlet crucian carp, locally called “Hibuna”, which shows unique scarlet body color variants, instead of the normal brownish silver color, at maturity and is found in several distant river systems on Hokkaido, the northernmost island of Japan [19]. Since a previous study reported diploidy and triploidy as the two main ploidy levels of Hibuna [20], we hereinafter refer to wild *Carassius* individuals showing a scarlet body color as “Hibuna” regardless of their ploidy. Particularly famous is the *Carassius* population including Hibuna in Lake Harutori, a coastal lagoon (surface area of 0.36 km^2^ and average water depth of 2.3 m) in Kushiro, Hokkaido [21]. This habitat of Hibuna has been designated as a Natural Monument of Japan since 1937 [20]. Since then, Hibuna has been considered as a symbolic fish of this lake and other watersheds in Hokkaido [20, 22]. However, a dramatic decline in the number of Hibuna in the lake has recently been reported, with no individuals being observed since the year 2000 in regular monitoring, which could be due to degradation of the lake environment caused by the deterioration of water quality and spawning grounds [21]. Fortunately, we had the rare opportunity to catch Hibuna in 2014 and 2015. To determine the appropriate conservation strategy for this special fish, there is a need to investigate its genetic characteristics and diversity.

Despite its symbolic status, the possible artificial origin of the Hibuna in Lake Harutori has been disputed by the recorded introduction of about 3,000 goldfish into the lake in 1916, before the first report of Hibuna in 1922 [21, 22]. Although the genetic characteristics of the Hibuna are largely unknown, the allozyme band pattern of diploid Hibuna was shown to be identical to that of goldfish, whereas that of triploid Hibuna was intermediate between those of goldfish and the co-habiting ordinary triploid *Carassius* [23]. The genetic difference between goldfish and Hibuna as well as the assumed reproductive separation of triploid Hibuna due to its clonal reproduction has been considered to provide evidence that Hibuna originated from *de novo* mutations. Then, at least triploid Hibuna has been inferred to be derived from *de novo* mutations in the pigmentation-related genes of the triploid *Carassius* that result in the unique scarlet body color of Hibuna [21, 23]. However, the results from a recent study on the genetic composition of Japanese triploids contradicted this assumption, by showing that unidirectional nuclear and mitochondrial gene flow from sexual diploids to triploids can occur in the *C*. *auratus*-complex [11]. This interploidy gene flow is probably derived from the recurring offspring of gynogenetic triploids that may result from mating between sexual female diploids and tetraploid males, which are produced by rare fertilization of triploid eggs with the sperm of a diploid male [11, 24, 25]. However, crossing experiments to confirm this interploidy gene flow have not yet been reported. There is thus a need to determine whether Hibuna is derived from *de novo* mutations or goldfish, and to investigate its genetic composition with reference to goldfish and other indigenous ordinary triploids.

In this study, the ploidy and genetic characteristics of the scarlet crucian carp, Hibuna, collected from Lake Harutori and the Abashiri River, which flows into the Sea of Okhotsk in Hokkaido, as one of the other known habitats of Hibuna [19], were investigated. This investigation compared the genetic characteristics of Hibuna with those previously reported for surrounding wild populations in Hokkaido and goldfish. We demonstrated the role of goldfish in the origin of triploid Hibuna, and the unexpectedly high genetic diversity in Hibuna, which could probably be attributed to repeated interploidy gene flow from goldfish to indigenous gynogenetic triploids.

## Materials and methods

### Ethics statement

Lake Harutori, the habitat of Hibuna, has been listed as a Natural Monument of Japan since 1937, where fish sampling is prohibited. Thus, we acquired permission for sampling from Kushiro City and Hokkaido Prefectural governments. For conservation reasons, we performed non-destructive sampling, with fish being released back into the lake after collecting a small amount of fin tissue, following a careful procedure. All procedures were conducted in accordance with the Guidelines for Proper Conduct of Animal Experiments [26], ARRIVE guidelines [27], and approved by the Animal Experimentation Committee of Kyoto University.

### Hokkaido *Carassius* populations and sampling design

**Fig 1:**
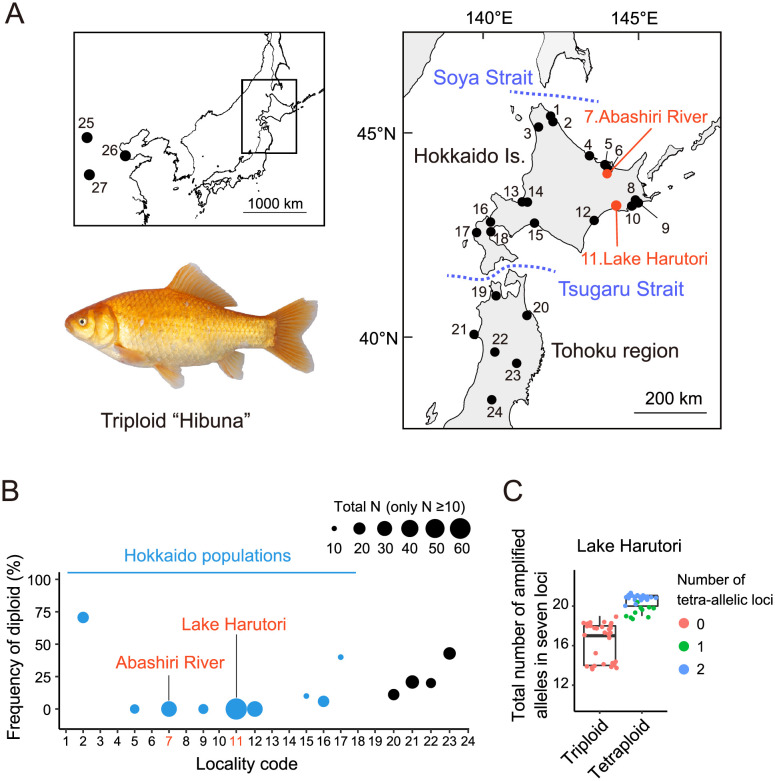
Sampling sites and estimated ploidy. (A) Sampling sites of the scarlet body color variants of *C*. *auratus*-complex, Hibuna (#7 and #11), and previously analyzed specimens reused in this study [11]. The map was generated from the R package mapdata [35]. (B) Frequency of diploids in each locality. Localities with a total number of specimens ≥10 are shown. The size of each point indicates sample size and Hokkaido populations are colored blue. (C) The total number of amplified alleles in seven microsatellite loci from triploid and tetraploid individuals in Lake Harutori. Each point indicates each specimen with color corresponding to the number of tetra-allelic loci.

Hokkaido Island is the northernmost Japanese island, separated from Honshu Island by Tsugaru Strait (130–140 m in depth) and from Sakhalin Island by Soya Strait (50–60 m in depth) (Fig 1A). The freshwater fish fauna on Hokkaido mainly includes species derived from Sakhalin Island through Soya Strait, which has repeatedly featured land bridges formed during glacial periods when lower sea levels have prevailed [28–30]. In contrast, Tsugaru Strait is considered to have been a significant barrier to the movement of freshwater fish due to its near-equivalent depth to the lowered ocean surface levels of 80–140 m during the glacial periods, and no firm land bridge has developed there since the middle Pleistocene [30–32]. However, several recent studies suggested southward migration of freshwater animals across this strait [33, 34]. Although *C*. *auratus*-complex populations in Hokkaido are believed to be derived from Honshu Island [29], it is still unclear whether they are indigenous or not. Recent extensive phylogeographic study, however, reported that the genetic characteristics of Hokkaido populations are close to those of northern Honshu (Tohoku region), but with distinct private mitochondrial haplotype groups, suggesting previous northward colonization of this species complex across Tsugaru Strait [11].

To test the role of goldfish in the origin of Hibuna, we referenced previously reported genetic characteristics of goldfish. Wang et al. (2013) reported mitochondrial gene genealogy of goldfish, covering the range of goldfish strains in China and Japan [36]. Their results showed that the mitochondrial haplotypes detected among goldfish strains were found in the Eurasian *C*. *auratus*-complex lineages referred to as clades C5 and C6, which are widespread in mainland China [36, 37].

Using this population background history to test the “goldfish origin hypothesis” for Hibuna, we first compared the mitochondrial haplotype of Hibuna with the mitochondrial sequences of goldfish deposited in a DNA database [36]. In addition, we compared the nuclear genetic characteristics of Hibuna with those of previously reported wild *C*. *auratus*-complex specimens [11] collected on Hokkaido, in Tohoku, and in the Yellow River of mainland China (Fig 1A).

### Ploidy determination

A total of 99 new samples, including 57 ordinary-colored *C*. *auratus*-complex and 42 Hibuna specimens, were collected from Lake Harutori (61 specimens, including 3 captive and 2 breeding stock Hibuna specimens) and the Abashiri River (38 specimens) on Hokkaido using a hand net and a cast net (Fig 1A, S1 Table). The analyzed Hibuna specimens collected from the Abashiri River were deposited at Bihoro Museum (seven specimens: BIHM0100925, 0100929, 0100948–0100952). Total genomic DNA was extracted from ethanol-fixed tissue specimens using the Wizard Genomic DNA Purification Kit (Promega, WI, USA). The ploidy of specimens was estimated by the number of alleles amplified from seven microsatellite loci (J12, GF17, GF1, J01, HLJYJ094, Cca19, and Cca12), which showed >97% consistency with the flow cytometry analysis conducted on two geographically distant populations [38]. It was effective for specimens collected from wide areas of Japan [11]. Simultaneously, it also provides information on the genetic relationships among individuals, such as clonality [38]. Genotypes were scored after the electrophoresis of amplification products with the size standard ROX 400HD or LIZ 500 on an ABI 3130xl sequencer (Applied Biosystems, CA, USA) using GeneMapper software 3.0 (Applied Biosystems). Previously reported genotypic data and ploidies of *Carassius* collected from Hokkaido, Tohoku, and the Yellow River in China [11] were combined for further population genetic analyses (S1–S3 Tables).

### Mitochondrial gene sequence analysis

Partial gene regions of mitochondrial cyt*b* (885 bp) were sequenced for all of the newly collected specimens. A primer pair, IF-f_L14724 and IF-r_H15915 [11], and GoTaq Green Master Mix (Promega) were used to conduct polymerase chain reactions (PCR). The PCR conditions were as follows: initial denaturation at 95°C for 120 s, followed by 30 cycles of amplification (95°C for 30 s, 48°C for 30 s, and 72°C for 60 s), and final extension at 72°C for 5 min. PCR products were treated with ExoSap-IT (USB Corp., OH, USA) at 37°C and then sequenced on an ABI 3130xl sequencer (Applied Biosystems) after the dye terminator reaction (BigDye^™^ Terminator Cycle Sequencing FS Ready Reaction Kit v3.1, Applied Biosystems) using the primer IF-f [11]. All mitochondrial haplotype sequences have been deposited in the International Nucleotide Sequence Database Collaboration (INSDC) (S3 Table; with accession nos.: cyt*b*, LC699673–LC699680). Previously reported sequence data from wild Japanese *C*. *auratus*-complex populations collected from Hokkaido and Tohoku [11] and those of goldfish haplotypes were downloaded [36] (S1 and S3 Tables). Mitochondrial haplotype nucleotide diversity was calculated using Arlequin v3.5 [39] for diploid and triploid/tetraploid populations with at least five specimens. Two breeding stock Hibuna specimens derived from Lake Harutori were excluded from this analysis. A maximum likelihood (ML) tree was constructed using a total of 224 unique haplotypes of *C*. *auratus-*complex and two haplotypes of sister species, *C*. *cuvieri*, as an outgroup using RAxML [40] with 1,000 bootstraps under the GTRCAT model. We labeled each cluster following the previously identified mitochondrial clades [11, 37].

### Nuclear genetic analysis

Nuclear genetic similarity between Hibuna and wild *C*. *auratus*-complex specimens was assessed based on genotypes from the seven microsatellite loci. The pairwise genetic distance between individuals was calculated using Bruvo’s distance [41] implemented in the R package POLYSAT [42]. This method considers the ambiguity in copy number of alleles in polyploids and is effective in populations with mixed ploidy. The resulting distance matrix data was used to construct the neighbor-joining (NJ) tree in the R package APE [43].

## Results

### Ploidy of Hibuna and ordinary crucian carp

Triploidy and tetraploidy were the main ploidies observed in the Hibuna collected from Lake Harutori and the Abashiri River, with the former location having 6 triploids and 29 tetraploids, and the latter having 7 triploids (Fig 1B and 1C; S1 Table). For the majority of tetraploids (66%) in Lake Harutori, their tetraploidy was supported by two tetra-allelic loci. In addition, all tetraploid specimens showed a larger total number of amplified alleles (19–21 alleles) in seven microsatellite loci than triploids in the lake (14–19 alleles; Fig 1C), supporting the accuracy of inferring tetraploidy. We found dominance of triploids in ordinary crucian carp collected from Lake Harutori (all 26 specimens were triploids) and the Abashiri River (of 37 specimens, 36 triploids and 1 tetraploid). Notably, no diploids were observed among the tested fish specimens. Overall, triploidy was the dominant ploidy in the Hokkaido *Carassius* populations, with it being exhibited by >80% specimens in seven out of nine localities (N ≥ 10), especially in the eastern Hokkaido populations, where no diploid individuals were found (Fig 1B, a total of 179 specimens from nine localities: Locality code #4–12), suggesting their rarity in this region.

### Genetic characteristics of mitochondrial DNA among Hibuna

Five haplotypes were detected from Hibuna using 885-bp cyt*b* mitochondrial sequences including five haplotypes in Lake Harutori, with one haplotype shared with the Abashiri River. The highest nucleotide diversity of 0.022 was observed in the mixed ploidy (triploid and tetraploid) group of Hibuna specimens from Lake Harutori, which was higher than the observed average nucleotide diversity of 0.0061 of 11 analyzed wild non-Hibuna populations from Hokkaido (S4 Table).

**Fig 2:**
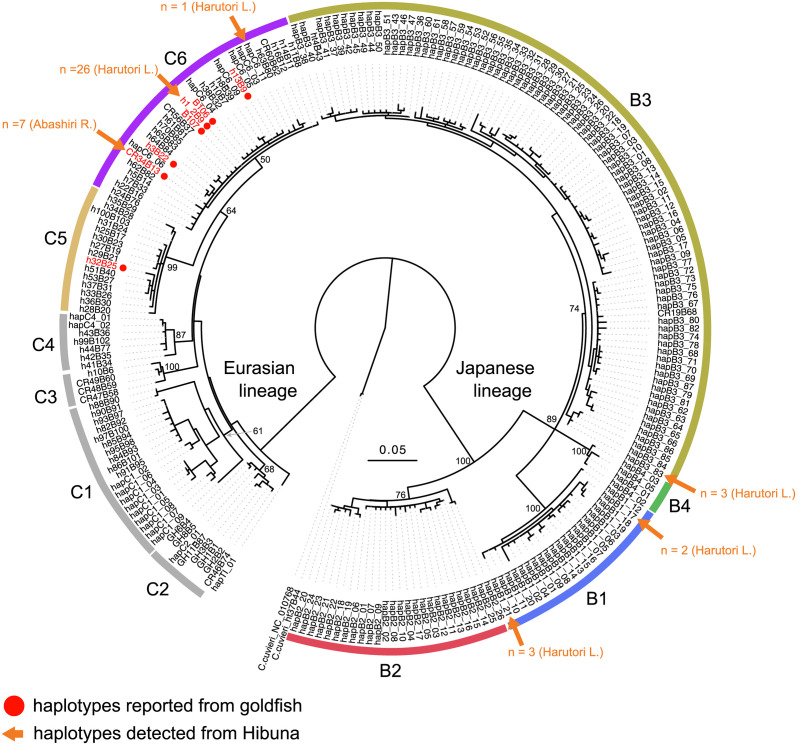
ML tree based on 885-bp sequences of mitochondrial cyt*b* region from 225 unique haplotypes of *Carassius* constructed using RAxML. The mitochondrial haplotypes previously reported from goldfish are colored in red circles. Scarlet arrows indicate haplotypes detected from Hibuna.

The ML tree was inferred to test for the involvement of goldfish in the origin of Hibuna using the reported mitochondrial haplotypes of goldfish. The results showed that the mitochondrial haplotypes detected from Hibuna were grouped into three divergent mitochondrial clades, including two Japanese lineages B1 and B3, and a Eurasian lineage C6. Overall, 81% of Hibuna specimens (42 specimens) showed mitochondrial haplotypes that were identical or very similar to previously reported goldfish haplotypes (number of specimens from Lake Harutori: no triploids and 27 tetraploids; the Abashiri River: 7 triploids; Fig 2), suggesting the genetic contribution of goldfish to Hibuna. This contribution was probably associated with the introduction of scarlet body color variants. These goldfish-like mitochondrial haplotypes were not common in the natural populations of Hokkaido Island, with the exception of two specimens of ordinary-colored triploid *Carassius* collected from the Abashiri River. Some Hibuna specimens (8 from Lake Harutori) showed regional mitochondrial haplotypes, which were also found in other wild Hokkaido populations and included in Japanese clades B1 and B3 (Fig 2; S1 Table).

### Nuclear genetic relationships

**Fig 3:**
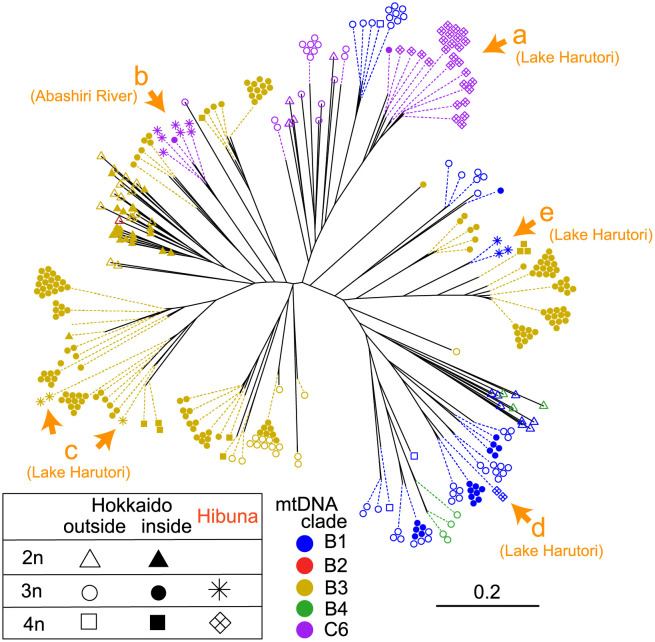
NJ tree based on the genetic distance between 358 individuals constructed using Bruvo’s distance obtained from seven microsatellite loci. For the specimens collected on Hokkaido including Hibuna, triploid and tetraploid individuals of identical genotypes were aligned on the corresponding tips. Clusters of Hibuna specimens are labeled as a–e with their sampling locality in orange. The triangular, circular, and square shapes at the tip represent diploid, triploid, and tetraploid, respectively, whereas the open or closed symbols indicate sampling regions outside and inside of Hokkaido, respectively. Asterisks and diamond crosses represent triploid and tetraploid Hibuna, respectively. The colors of symbols correspond to mitochondrial lineages of specimens.

The NJ tree constructed from the inter-individual genetic distance of seven microsatellite genotypes mostly showed clusters that were concordant with the mitochondrial background of specimens (clades B1, B3, and C6; Fig 3). In most of the triploid and tetraploid specimens, including triploid and tetraploid Hibuna, we observed other individuals with identical or very similar genotypes, indicating that they reproduce clonally via gynogenesis. These clonal lineages of Hibuna were found in at least five divergent clusters, and Hibuna collected from Lake Harutori and the Abashiri River were differently grouped (clusters a–e; Fig 3). Although some clustered with the *Carassius* specimens from China or were intermediate, showing inconsistent mitochondrial background and nuclear genetic relationships (Fig 3), two lineages with mitochondrial haplotypes belonging to the B1 or B3 clade formed a cluster with wild populations on Hokkaido Island.

## Discussion

### Ploidy and reproduction of Hibuna

We detected triploidy or tetraploidy as the main ploidies of the scarlet *Carassius* fish, Hibuna, making this the first record of tetraploidy among them. Tetraploids are generally rare in wild *Carassius* populations [11, 24, 38] and probably result from fertilization of unreduced triploid female (3n) eggs with sperm (n) donated from a diploid male [25]. Previous characterization of progeny between male or female tetraploid and diploid *Carassius* in Japan and Eurasia showed that female tetraploids reproduce gynogenetically, whereas crossing between male tetraploids and female diploids produces triploid offspring [24]. Consistent with this, the seven microsatellite markers revealed that several pairs among tetraploid (and triploid) Hibuna had identical genotype, suggesting that at least some of the tetraploid Hibuna reproduce gynogenetically in the wild. The establishment of such gynogenetic tetraploidy is assumed to have contributed to the high incidence of tetraploid *Carassius* in Lake Harutori.

Similar increases in ploidy levels resulting from sperm nuclear incorporation have been reported in several gynogenetic vertebrate species, such as *Poecilia* fish, Amazon molly, and unisexual salamander in the genus *Ambystoma* [44]. Although the sex and reproductive mode of these species remain poorly characterized, the tetraploid *Ambyostoma* unisexuals have been shown to have a higher rate of developmental defects and greater larval mortality than triploids, probably due to the misconfiguration in parthenogenetic egg development by ploidy elevation [44–46]. The occurrence of both male and female tetraploid *Carassius* exhibiting different reproductive modes could be a unique characteristic among asexual vertebrates. This finding provides a valuable clue for future studies on the cytological mechanisms of gynogenesis in this fish complex.

### Origin of Hibuna

The “*de novo* mutation” and “goldfish-derived” hypotheses have been proposed to explain the origin of Hibuna [21–23]. Goldfish-like mitochondrial haplotypes were observed in Hibuna collected from Lake Harutori and the Abashiri River, but were uncommon among other populations from Hokkaido. The record shows that about 3,000 goldfish were artificially introduced into Lake Harutori in 1916 [22]. In addition, nuclear and mitochondrial gene flow from sexual diploid to gynogenetic triploid *Carassius* has been reported for samples from all over Japan [11]. Taken together, these findings suggest that Hibuna is a hybrid of the native triploids and artificially introduced goldfish (or their descendants, probably including hybrids formed from crossing with native diploids).

**Fig 4:**
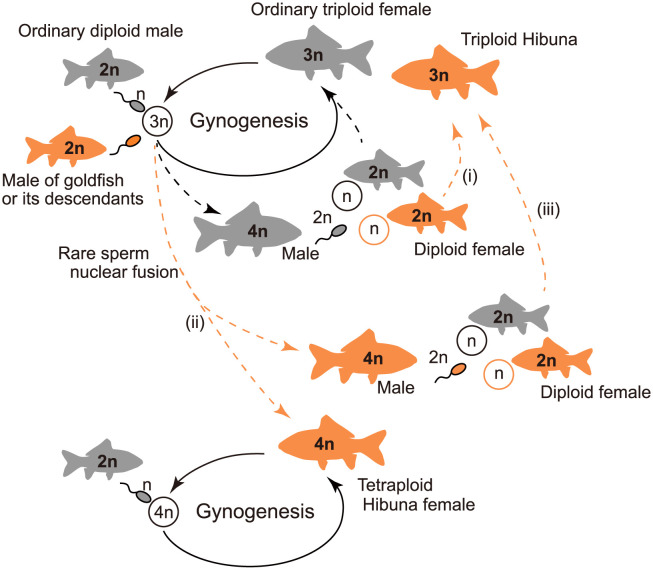
The expected scenario for the origin and diversification of Hibuna. Gray- and scarlet-colored fish symbols indicate ordinary and scarlet body color variants of *Carassius* fish, respectively. Scarlet dashed lines represent likelihood of introgression from goldfish to triploid lineages.

The introduction of the scarlet body color variant’s allele(s) from goldfish (or their descendants) to the triploid lineage and maintenance of the alleles are likely to have occurred via the following scenarios (Fig 4): (i) Hybridization between a tetraploid male, which produces diploid sperm [24, 47], and a female goldfish (or its descendants) results in triploids with the body color variant. (ii) Fertilization of unreduced eggs (3n) of a triploid with sperm (n) from a male goldfish (or its descendant) results in tetraploid male or female offspring with the body color variant. The resulting female tetraploids in this scenario are expected to reproduce gynogenetically, whereas (iii) the male tetraploids (producing 2n sperm) would likely hybridize with diploid females (producing n eggs) and produce the scarlet triploid fish. When these hybridization processes involved female diploids or triploids having an indigenous mitochondrial haplotype, it can be attributed to the possession of indigenous mitochondrial haplotypes in Hibuna. Although we cannot completely rule out the “*de novo* mutation hypothesis” regarding the origin of Hibuna, the “goldfish-derived hypothesis” can simply explain the occurrence of the scarlet body color variant in eastern Hokkaido and the genetic similarity of Hibuna to goldfish.

### Implications for conservation

Hibuna is a symbolic fish and its habitat (Lake Harutori) was designated as a Natural Monument of Japan in 1937. This study revealed that Hibuna was derived from hybridization between introduced goldfish (or their descendants) and native gynogenetic triploid *Carassius* via rare sexual reproduction. The observed high genetic diversity among Hibuna strongly suggests that genetic diversity of sexual relatives would have contributed to the genetic diversity of gynogenetic triploid *Carassius*, as recently shown for Japanese populations [11]. Thus, Hibuna is a valuable example of a cross between sexual diploid and gynogenetic triploid in the wild in a short time (<100 year) and highlights the unique evolutionary features of triploid *Carassius*. However, considering the negative aspect of genetic disturbance regarding the origin of Hibuna, when considering their priority for conservation, the preservation of other wild surrounding populations in eastern Hokkaido needs to be taken into account. Additionally, it is necessary to prevent the further spread of Hibuna or the introduction of goldfish into natural waters. Fortunately, in the case of Lake Harutori, it is expected that the dispersal of Hibuna is limited and can be managed because of their protection by law and the small river basin size (basin area: 5.3 km^2^, stream length: 4.6 km).

The conservation of gynogenetic species is complicated by both their dependence on and competition with their sexual relatives. A previous study on the frequency of triploids of *Carassius* in 24 localities across Hokkaido in 1977–1981 reported that triploids were dominant in most localities (18 out of 24 locations) [48]. Thirty years later, we also observed the pronounced dominance of triploids or tetraploids. These results suggest serious challenges in the conservation of Hibuna and other wild Hokkaido populations, especially populations in eastern Hokkaido, where sexual diploid *Carassius* was at a very low frequency (not observed). Some of these diploid and triploid *Carassius* populations might already be at risk of extirpation. Considering the need for sexual diploids to maintain triploids, the decline of diploids has probably occurred recently. Although the causes of such a very low frequency of diploids are unknown, their fitness may have been decreased by anthropogenic impacts such as deterioration of the quality of their spawning grounds and habitat [21], which might negatively affect diploids more and intensify competition between diploids and triploids. Thus, to develop effective conservation policies for protecting the set of ploidies of *Carassius*, there is a need to identify the actual cause of the population decline and clarify the factors contributing to their stable coexistence. With respect to the latter, the leading explanations are lower parasite resistance [49, 50] and inferior aerobic performance of triploids (and probably tetraploids), the latter of which is assumed to be due to the lower number and oxygen-carrying capacity of erythrocytes in triploids [51, 52]. In addition, since the Japanese diploids are genetically distinct from the triploid *Carassius* (i.e., a hybrid between Japanese and Eurasian lineages [11]), some ecological or reproductive differences between ploidies might help their coexistence by alleviating competition between them [12, 53].

Another possibility for explaining the maintenance of triploids despite the very low frequency or absence of sexual diploids is that triploids might avoid the risk of extirpation by acquiring parthenogenesis or by changing the species of the sexual sperm donor. However, evolution to sperm-independent parthenogenesis has not yet been observed in any animals [5]. The switching of sperm donor has been reported in several gynogenetic species, including *Poecilia* fish [54, 55], *Ambystoma* salamander [45], and *Cobitis* loach [56, 57]. In Eurasian triploid *Carassius*, several studies reported the existence of triploid males [58, 59], although most of the previous intensive studies failed to find any triploid males in Japan [17, 60–63]. However, considering the reported temperature-dependent sex differentiation in diploid *Carassius* [64–66], such accidentally occurring rare triploid males might contribute to gynogenetic reproduction as sperm donors. Additionally, the gynogenetic triploid *Carassius* has shown the ability to use divergent lineages in the diploid *C*. *auratus*-complex as a sperm donor [11, 37], and artificial fertilization experiments have shown the capacity for their development with sperm from ranges of other cypriniform fishes such as carp and loach [67–69]. Thus, we cannot rule out the use of sperm donors other than diploid *Carassius* by Hokkaido triploid populations, where sexual diploid *Carassius* is rare or absent. This possibility should be confirmed by ecological investigation on the reproduction of the triploids.

In summary, our study revealed that the artificial origin of Hibuna involved hybridization with goldfish. The unintended 100-year field experiment after the introduction of goldfish into the lake exemplified various reproductive modes and complex diversification processes occurring in *Carassius* fish via interploidy gene flow involving rare sexual reproduction. Thus, Hibuna’s unique scarlet body color symbolizes the complexity of reproduction among vertebrates. However, the priority to be placed on the conservation of Hibuna relative to that of the surrounding populations should be reconsidered because the observed dominance of triploids in most Hokkaido *Carassius* populations implies a hidden risk of their co-extirpation. There is thus a need for studies on ecological and physiological differences between the diploid and triploid fish in order to establish appropriate conservation policies for the *Carassius* populations, especially the diploids, on Hokkaido. The maintenance of sexual diploid populations will also stabilize triploid populations by providing sperm donors and facilitating the genetic diversity of triploids via interploidy gene flow.

## Supporting information

S1 Table Sampling locations, number of individuals and mitochondrial haplotypes of *Carassius* fish obtained from Abashiri R. and Lake Harutori (colored in red) and surrounding populations (Mishina et al. 2021). (XLSX)

S2 Table Genotypes for *Carassius* fish obtained by 7 microsatellites. (XLSX)

S3 Table Accession numbers of mitochondrial cytb sequences. Accessions with identical sequences to the one from other accessions are shown in parentheses.
(XLSX)

S4 Table Mitochondrial nucleotide diversity based on partial cytb region of natural triploid/tetraploid populations in Hokkaido and Tohoku regions, Japan. The statistics of Hibuna populations are shown in bold.
(XLSX)
